# Metal-Based Nanoparticles and the Immune System: Activation, Inflammation, and Potential Applications

**DOI:** 10.1155/2015/143720

**Published:** 2015-06-01

**Authors:** Yueh-Hsia Luo, Louis W. Chang, Pinpin Lin

**Affiliations:** ^1^Division of Environmental Health and Occupational Medicine, National Health Research Institutes, 35 Keyan Road, Zhunan 35053, Miaoli County, Taiwan; ^2^National Environmental Health Research Center, National Health Research Institutes, 35 Keyan Road, Zhunan 35053, Miaoli County, Taiwan

## Abstract

Nanomaterials, including metal-based nanoparticles, are used for various biological and medical applications. However, metals affect immune functions in many animal species including humans. Different physical and chemical properties induce different cellular responses, such as cellular uptake and intracellular biodistribution, leading to the different immune responses. The goals of this review are to summarize and discuss the innate and adaptive immune responses triggered by metal-based nanoparticles in a variety of immune system models.

## 1. Introduction

Nanotechnology is one of the most exciting industrial innovations of the 21st century. Nanomaterials are used in various industrial applications and products, including sporting goods, tires, sunscreens, cosmetics, electronics, and fuel additives as well for a variety of medical purposes such as diagnostic imaging and drug delivery. Many nanomaterials are metal-based nanoparticles, such as nanosilver, nanometallic oxides (zinc oxide, titanium dioxide, iron oxide, and quantum dots), and are applied for many uses [1]. For example, zinc oxide (ZnO) and titanium dioxide (TiO_2_) are used in sunscreens and cosmetic products [2, 3], and nanosilver is used in detergents, antibacterial agents, paints, printer inks, and textiles [4–9].

Nanoparticles frequently have remarkably different physicochemical properties than their conventional bulk materials. These properties can be a “double-edged sword,” providing positive advantages for usefulness and negative impacts on health upon exposure. Toxicity due to some metal-based nanoparticles such as silver, gold, and copper increased with decreasing nanoparticle size [10]. Other physicochemical properties such as elemental composition, charge, shape, crystallinity, surface area, solubility, and surface derivatives also influence the toxic potential of the compounds [11–15]. Therefore, metal-based nanoparticle should not be considered a homogeneous population with simple toxic attributes because they act independently to mediate diverse biological reactions.

Many investigators have explored the properties and toxicities of various metal-based nanoparticles. The toxicities of various metal-based nanoparticles, both* in vitro* and* in vivo*, were recently reviewed and summarized by Schrand et al. (Table 1) [10].

**Figure 1:**
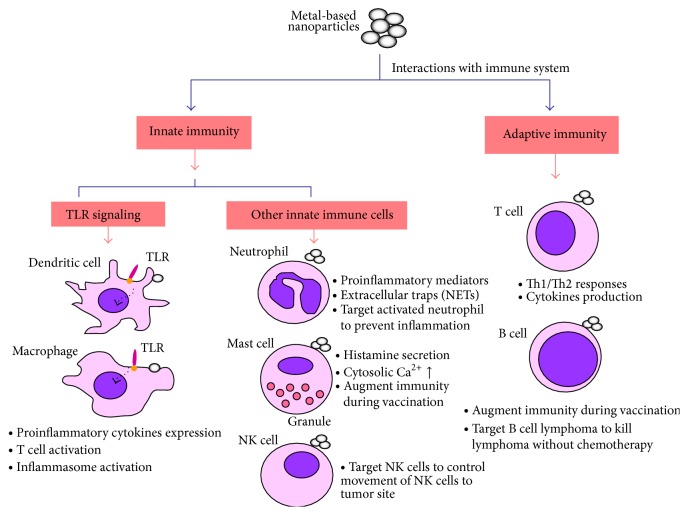
Metal-based nanoparticles interaction with immune system.

The engineering of nanoparticles for application in the immune system is now an exciting, emerging field. Although certain nanomaterials are immunotoxic or immunomodulatory, a concise overview of the interactions between nanoparticles and the immune system would be valuable and indispensable to students and researchers alike. The focus of this review is to outline the interactions of innate and adaptive immune systems with metal-based nanoparticles (Figure 1). We discuss the role of toll-like receptors interaction with nanoparticles and their potential implications. Different effects of nanoparticles on innate immune cells (macrophages, dendritic cells, neutrophils, mast cells, and natural killer cell) and adaptive immune cells (T cells and B cells) are reviewed. This information will enhance the understanding for immunological effects of nanomaterials and help to develop safe metal-based nanoproducts.

## 2. Nanoparticles and Immune System

The immune system can defend against foreign antigens, which has been divided into two general types of immunity: innate immunity and adaptive immunity. Innate immunity is the nonspecific and first line of the body's defense system, which relies on pattern recognition receptors (PRPs) to recognize broad and conserved molecular patterns found on pathogens (pathogen-associated molecular patterns, PAMPs) [16]. Therefore, the innate immune system plays an essential role in the early recognition and subsequent proinflammatory response. The adaptive immune system is antigen specific and reacts only with the organism that induced the response. Innate and adaptive immunity can be thought of as two equally important aspects of the immune system.

Most nanoparticles are recognized as foreign materials and eliminated by the immune system. However, in the immune system, if the foreign materials are not recognized as a threat, they are ignored or tolerated. Undesirable overwhelming activation of immune responses may lead to harmful consequences. Therefore, the response of the immune system to the nanoparticles must be considered when developing a nanomaterial for* in vivo* application. For example, avoiding immune system detection is crucial if a nanomaterial is to be used for gene or drug delivery [17]. In contrast with avoiding immune system of nanoparticle drug delivery, nanoparticles also can play importing role in vaccine immunization via antigen delivery and adjuvanticity. Another viewpoint is that nanoparticles targeting immune cell (e.g., macrophages or dendritic cells) can manipulate or control immunological diseases such as infectious disease or tumor therapy. For example, nanomaterial might be designed to modify effective immune responses of tumor microenvironment via accompanied with anti-inflammatory drug or specific cytokines.

Three immune related consequences must be considered when a nanomaterial is engineered for application* in vivo*. The first is immune-mediated destruction or rejection, which could initiate a defensive immune reaction resulting in the elimination of the nanomaterials. Second is immunotoxicity, which could damage the immune system and cause pathological changes. The third is immunocompatibility, which does not interfere with the immune response [18]. Nanoparticle properties such as size, charge, hydrophobicity, hydrophilicity, and the steric effects of nanoparticle coatings direct nanoparticle compatibility with the immune system [17, 19, 20]. For example, nanoparticles that are designed by encapsulated PEG or other types of polymers provide a hydrophilic environment and shield them from immune recognition [21]. However, some reports showed that the immune system can produce PEG-specific antibodies after administration of PEG-coated liposomes [22, 23]. The researches focus on how and whether nanoparticles triggered antibodies production is limited and we need further studies to answer these inconclusive questions.

### 2.1. Nanoparticles and Innate Immunity

Innate immune system consists of different cells and proteins that are nonspecific and first line of defense system. The main components of the innate immune system are including physical epithelial barriers, phagocytic cells (monocyte/macrophages, dendritic cells, and polymorphonuclear leukocytes), phagocytic leukocytes, basophils, mast cells, eosinophils, natural killer (NK) cell, and circulating plasma proteins.

In recent decades, many studies have rapid progress in toll-like receptor of innate system, which induce expression genes of involved inflammation. Moreover, toll-like receptors activate both innate and adaptive immune system and play an important role in antiviral and anti-immunity [24]. In this review, we will first discuss the toll-like receptor signaling mechanisms triggered by metal-based nanoparticles and then describe the effects of nanoparticles on other innate immune cells.

### 2.2. The Role of Toll-Like Receptor Signaling in Innate Immune System

The innate immune system, also known as nonspecific immune system and first line of defense, relies on recognition of PAMPs through a limited number of germ line-encoded pattern recognition receptors, belonging to the family of toll-like receptors (TLRs) [25]. The* Toll* gene was originally discovered in* Drosophila*, responsible for dorsoventricular polarization during embryonic development and antifungal and antibacterial properties of the adult fly [26]. TLR1, TLR2, TLR4, TLR5, TLR6, and TLR10 are present on the cell surface whereas TLR3, TLR7, TLR8, TLR9, TLR12, and TLR13 are localized into intracellular vesicles such as endosomes, lysosomes, and ER. TLR1/TLR2 sense bacterial tri-acylated lipopeptides. TLR2/TLR6 recognize di-acetylated lipopeptides and bacterial lipoteichoic acid or peptidoglycans and mycobacterial cell wall components. TLR3 binds to viral double stranded RNA; while TLR4 responds to LPS, TLR5 senses flagellin. TLR7 and TLR8 respond to the single stranded RNA from viruses, while TLR9 binds to DNA-containing unmethylated CpG motifs which are commonly found in bacterial DNA. TLR12 recognizes profilin, while TLR13 senses bacterial 23S ribosomal RNA (rRNA) [16]. The activations of TLR signalings can not only induce cytokines production but also increase macrophages phagocytosis and natural killer (NK) cells cytolytic activity. Most importantly, TLR signaling activations also can enhance antigen presentation via upregulating the expression of major histocompatibility complex (MHC) and costimulatory molecules (CD80 and CD86) on dendritic cells leading to adaptive immunity activations. Thus, the TLR agonists were believed as powerful vaccine adjuvants, allergy, infection, and antitumor therapeutics in preclinical studies [24]. The TLR antagonists also have therapeutic values in clinical trial to treat septic shock and autoimmune [27]. For example, TLR agonists or nanoparticles that enhanced TLR signaling pathways would be powerful adjuvants [28, 29]. In contrast, TLR antagonists or inhibitors that reduced the inflammatory response would have beneficial therapeutic effects in autoimmune diseases and sepsis [30]. These potential applications may open up innovative directions for the design of nanoparticle conjugates to meet different requirements.

### 2.3. Effects of Nanoparticles on TLR Signaling of Innate Immunity

TLRs are classified as type I transmembrane receptors containing an N-terminal leucine-rich repeat domain (transmembrane region) and a C-terminal cytoplasmic domain. Upon recognition of a PAMP, TLRs recruit a specific set of adaptor molecules that contain the TIR domain, such as MyD88 and TRIF, and initiate downstream signaling events that lead to the secretion of inflammatory cytokines, type I IFN, and chemokines [31]. The TLR signaling cascade results in the activation of transcription factors, nuclear factor *κ* light chain enhancer of activated B cells (NF-*κ*B), interferon-regulatory factors (IRFs), and mitogen-activated protein kinase; these factors affect the transcription of genes involved in inflammatory and immune responses [32, 33].

Schmidt et al. first reported that Ni^2+^ as an inorganic activator was acting directly through TLR binding to trigger inflammation responses [34]. This interesting finding also makes us think of whether the other chemicals components such as metal-based nanoparticles were also involved in TLR signaling inflammation. Recently, several studies have demonstrated the effects of nanoparticles on innate immunity via TLR signaling pathways [35]. Several nanoparticles (e.g., TiO_2_, ZnO, zirconium dioxide (ZrO_2_), and silver) modulated immune responses via TLRs. TiO_2_ and ZrO_2_ nanoparticles increased* TLR7* and* TLR10* mRNA levels in human macrophage U-937 cells and* TLR2* and* TLR4* mRNA levels in the mouse liver cells [36, 37]. N-(2-Mercaptopropionyl) glycine (tiopronin) capped-silver nanoparticles enhanced the TLR3 ligand and TLR9 ligand-induced IL-6 secretion in mouse macrophage Raw264.7 cells [38]. ZnO nanoparticles induced MyD88-dependent proinflammatory cytokines via a TLR signal pathway [39]. Quantum dot 705 activated MyD88-dependent TLRs at the surface or inside of cells, which is a fundamental mechanism for nanoparticle-induced inflammatory responses [40]. TLRs may have important roles not only for different NPs uptake but also for their cellular response [41]. Moreover, the mechanisms of interaction between NPs and TLR are still unclear. There are two possibilities to explain how NPs interact with TLRs. One is that the smaller NPs may just like LPS have cooperated with some small molecules such as the LPS binding protein and then the complex activates further TLRs signaling pathways. The other is that the larger size of NPs may directly associate with TLRs [41]. However, these hypotheses need more studies to confirm.

Proinflammatory cytokines can be induced by TLR signaling pathways. Many cytokines, such as interleukin- (IL-) 1, IL-6, and tumor necrosis factor- (TNF-) *α*, can activate inflammatory cells, increase vascular permeability, and cause swelling and redness during acute inflammatory responses [42]. IL-1 and IL-6 are important mediators of fever [43]. TNF-*α* activates endothelial cells leading to hypotension. IL-8 is a chemokine that activates neutrophils or other granulocytes and recruits them to the site of inflammation [44]. Interferon- (IFN-) *γ* plays an important role in the inflammatory process, recruiting macrophages to the site where antigen is present [42]. Many studies have reported that NPs can trigger cytokines production which associated with inflammatory responses. The levels of proinflammatory cytokines are measured as biomarkers of nanoparticle immunomodulatory effects and immune-mediated toxicity [42]. TiO_2_ nanoparticles, nanodiamond, and nanoplatinum also are reported to trigger proinflammatory cytokine production, dendritic cell maturation, and naïve T cell activation and proliferation [45, 46]. Hanley et al. also reported that ZnO nanoparticles increased the expression of IFN-*γ*, TNF-*α*, and IL-12 in primary human immune cells [47]. Gold nanoparticles (10 nm and 50 nm in size) induced IL-1*β*, IL-6, and TNF-*α* in rat liver cells after 1 day of acute treatment and then subsided by day 5 of subchronic treatment. The 50 nm gold nanoparticles produced more severe inflammation than the 10 nm gold nanoparticles [48]. However, limited studies demonstrated whether or which TLR is involved in the NPs induced proinflammatory cytokines production.

Another interesting field is inflammasomes which are multiprotein complexes leading to caspase-1 activation, further causing pro-IL-1*β* and pro-IL-18 maturations and secretions. The IL-1*β* synthesis and secretion are tightly regulated by TLR signaling and inflammasome activation. A first signal, such as toll-like receptor activation, triggers synthesis of pro-IL-1*β* by transcriptional induction, whereas a second stimulus leads to inflammasome oligomerization, caspase-1 autoactivation, and caspase-1-dependent cleavage and release of the biologically active, mature IL-1*β*  [49]. The second signal can be triggered by an ever-expanding group of chemically and biologically unrelated danger-associated molecular patterns (DAMPs) or pathogen-associated molecular patterns (PAMPs) [50]. The study of nanoparticles that induce IL-1*β* via inflammasome signaling pathways mechanism is an emerging theme [51, 52].

Some engineered nanoparticles can also activate inflammasome signaling pathways [49, 53, 54]. Among various inflammasomes, nucleotide-binding oligomerization domain- (NOD-) like receptor protein 3 (NLRP3) activation is linked to exposure to various nanoparticles [54, 55]. TiO_2_ and SiO_2_ nanoparticles activate the NLRP3 inflammasome and IL-1*β* release in LPS-primed murine bone marrow-derived macrophages and human macrophage cell lines THP-1 [49, 56]. Peeters et al. [55] recently reported that crystalline silica (SiO_2_) activated NLRP3 inflammasomes in human lung epithelial cells BEAS-2B and primary human bronchial epithelial cells, which prolonged the inflammatory signal and affected fibroblast proliferation. Silver nanoparticles induced inflammasome formation and triggered IL-1*β* release and subsequent caspase-1 activation [53]. Inflammasome-activation-associated IL-1*β* production by dendritic cells in response to particle treatment was size-dependent and maximal at particle diameters between 400 and 1000 nm [57]. Yazdi et al. reported that nano-TiO_2_ and nano-SiO_2_, but not nano-ZnO, activate the NLRP3 inflammasome, leading to IL-1*β* release, and in addition induce the regulated release of IL-1*α*. Unlike other particulate NLRP3 agonists, nano-TiO_2_-dependent NLRP3 activity does not require cytoskeleton-dependent phagocytosis and induces IL-1*α*/*β* secretions in nonphagocytic keratinocytes. However, the exact mechanism of nano-TiO_2_ uptake remains elusive, as blocking lipid raft-mediated, caveolin-dependent, or clathrin-dependent endocytosis did not efficiently block IL-1*β* secretion [49]. The more knowledge we have of cytokine profiles induced by nanoparticles, the better we can utilize the cytokines as biomarkers of immunomodulatory properties of nanoparticles. Moreover, it is also necessary to clarify whether these proinflammatory cytokines were induced by nanoparticle physiochemical properties or by bacterial endotoxin contaminants.

### 2.4. Effects of Nanoparticles on Innate Immune Cells

The innate leukocytes include mast cells, neutrophils, eosinophils, basophils, natural killer (NK) cells, gamma/delta T cells, and the phagocytic cells including macrophages and dendritic cells. We summarized several studies which reported the effects of metal-based nanoparticles on phagocytic cells, neutrophils, mast cell, and NK cells. There are still many challenges to investigate the effects and potential applications of nanoparticles to other innate immune cells such as eosinophils, basophils, and gamma/delta T cells.

#### 2.4.1. Phagocytic Cells (Macrophages, Dendritic Cells)

Macrophages and dendritic cells play many key roles in host defense system. They can remove dead cells and pathogens by phagocytosis. They also can shape the inflammatory response by secreting cytokines through TLR signaling pathway and modulate adaptive immunity by presenting antigens to lymphocytes [58]. In general, macrophages and dendritic cells readily uptake nanoparticles. Therefore, many metal-based nanoparticles (e.g., magnetic nanoparticles and nanoparticles-based PET agents) were commonly used for visualizing of macrophages in human diseases including cancer, atherosclerosis, myocardial infarction, aortic aneurysm, and diabetes [58]. In addition to image applications, targeting tumor-associated macrophages or dendritic cells via nanoparticles for drug, antigen delivery, or vaccine is also a promising tumor therapeutic application. For example, Lin et al. reported that gold nanoparticle delivery of modified CpG can stimulate macrophages and inhibits tumor growth for immunotherapy [59]. Ahn et al. recently demonstrated that gold nanoparticles enable efficient tumor-associated self-antigen delivery to dendritic cells and then activate the cells to facilitate cross-presentation, inducing antigen-specific cytotoxic T cell responses for effective cancer therapy [60].

#### 2.4.2. Neutrophils

During acute inflammation, polymorphonuclear neutrophil cells (PMNs) are the first type of leukocytes to migrate to an inflammatory site and then produce several proinflammatory mediators including chemokines, which further attract other PMNs and other cell types like monocytes-macrophages and lymphocytes, corresponding to chronic inflammation. Gold nanoparticles were found trapped by neutrophils in their extracellular traps (NETs), being composed mainly of DNA and a variety of antibacterial proteins [61]. The cell-gold networks were visible after as early as 15 min of treatment of neutrophils with the gold nanoparticles. NETs may contribute to alerting the immune system of a danger signal by activating DNA receptors such as TLR9. This activation might turn out to help in the recruitment of immune cells to mount an acquired immune response or to resolve the inflammation. NETs can either fight inflammatory disease or cause disease depending on the place, time, and dose [62]. However, NETs triggered by nanoparticles need further investigation to figure out their physiological roles. Wang et al. found that delivery of drugs into inflammatory neutrophils by nanoparticles can prevent vascular inflammation [63]. This study provides a novel nanoparticle-based therapeutic approach for targeting activated neutrophils to treat a range of inflammatory disorders.

#### 2.4.3. Mast Cells

Mast cells contain many granules in histamine and heparin and have important roles of allergy and anaphylaxis. When activated, mast cells rapidly release histamine and heparin from their granules to dilate blood vessels and recruit neutrophils and macrophages. Chen et al. demonstrated that TiO_2_ nanoparticles not only dose-dependently increased histamine secretion, but also increased cytosolic Ca^2+^ concentration in rat mast cells [93]. Their results suggest that systemic circulation of nanoparticles may prompt histamine release without prior allergen sensitization, causing abnormal inflammatory diseases or potential exacerbating manifestations of multiple allergic responses. It is recently reported that the granules of mast cells are powerful enhancers of adaptive immunity when they are released at sites of infection or vaccine administration. John et al. engineered nanoparticles consisting of mast cells granules to augment immunity during vaccination [94]. It is believed that other metal-based nanoparticles also have possibility of developing this efficient vaccination system.

#### 2.4.4. NK Cells

NK cells control several types of tumors and microbial infections by limiting their spread and subsequent tissue damage. NK cells are also regulatory cells which can interact with dendritic cells, macrophages, T cells, and endothelial cells. Therefore, NK cells are believed that they can limit or exacerbate immune responses [95]. Clinical study has demonstrated that patients with a high level of NK infiltration were found to have a better prognosis than those with a low level of NK infiltration and suggests that enhancement of NK cell infiltration could be a useful antitumor strategy [96]. Lim et al. provided evidences of cell tracking with quantum dots (QD) by labeling NK cells with anti-CD56 antibody-coated QD705 and tracking the labeled cells up to 12 days after intratumoral injections [97]. The authors further found a decreased size of tumors treated with NK cells compared with controls [97]. QD labeling was thought as the well-suited imaging technique for tracking different cell populations; however, currently available compounds are not clinically applicable because of toxic cadmium cores or other nondegradable components; cadmium-free or biodegradable QDs are currently being developed [98]. Jang et al. used magnetic nanoparticles (Fe_3_O_4_/SiO_2_) to control movement of human natural killer cells (NK-92MI) by an external magnetic field, loading NK-92MI cells infiltrated into the target tumor site and their killing activity is still maintained the same as the NK-92MI cells without the nanoparticles [99]. This study provides an alternative clinical treatment with reduced toxicity of the nanoparticles and enhanced infiltration of immunology to the three-dimensional target site without surgical treatment.

### 2.5. Nanoparticles and Adaptive Immunity

Nanoparticles can be designed to deliver vaccine antigens through specific intracellular pathways such as phagocytosis, macropinocytosis, and endocytosis, allowing better antigen presentation for activating the adaptive immune system [100]. Nanoparticles interact most frequently with APCs in the blood circulation, including B cells, macrophages, and dendritic cells. APCs engulf and digest foreign antigens present on the surface major histocompatibility complexes of B and T cells [101]. Dendritic cells are the most specialized APCs, which capture and process antigens and migrate to lymphoid tissues leading to T cell or B cell activation. The costimulatory molecules of dendritic cells and the cytokine environment affect the T cell response. T cells including T helper (Th) cells, regulatory T cells (formerly known as suppressor T cells), and cytotoxic T cells express various surface proteins including CD3 and CD4 on Th cells, CD3, and CD8 on cytotoxic T cells. The cytokine environment is produced by dendritic cells via activated CD4+ T cells, neutrophils, and macrophages, which are recruited to the inflammatory site and stromal cells [100]. For example, immature dendritic cell encountered antigens, which are presented to T cells for self-tolerance (T cell anergy) without costimulatory molecule expression. This also occurs for regulatory T (Treg) cells in the presence of transforming growth factor-*β*1 (TGF-*β*1) and interleukin- (IL-) 10. Exogenous antigen activates and matures dendritic cells leading to costimulatory molecule expression and Th1, Th2, or Th17 cell activation [102]. Antigen presentation in the IL-6 and IL-23 cytokine microenvironment can also stimulate naïve CD4+ T cells to differentiate into Th17 cells [100]. Th17 cells are potent inducers of inflammation and play key roles in the development of autoimmunity diseases [103]. Th1 cells mediate cellular immunity and further regulate inflammation responses. On the other hand, Th2 cells induce proliferation of master cells and eosinophils and mediate the differentiation of B cells to produce immunoglobulin (Ig) G and IgE, thereby promoting humoral immunity [42].

### 2.6. Effects of Nanoparticles on T Cells

Only several metal-based nanoparticles were reported to activate T cell responses or homeostasis. For example, TiO_2_ nanoparticles provoke inflammatory cytokines and increase dendritic cell maturation, expression of costimulatory molecules, and prime naïve T cell activation and proliferation [45]. Cd trapped inside fullerene cage nanoparticles (Gd@C82(OH)22) has specific immunomodulatory effects on T cells and macrophages, including polarization of the cytokine balance towards Th1 cytokines, decreasing the production of Th2 cytokines (IL-4, IL-5, and IL-6) and increasing the production of Th1 cytokines (IL-2, IFN-*γ*, and TNF-*α*) [104]. One important theory of adaptive immunity is T cell homeostasis (Th1/Th2 balance). Th1 cells drive the cellular immunity to fight viruses and other intracellular pathogens, eliminate cancerous cells, and stimulate delayed-type hypersensitivity skin reactions. Th2 cells drive the humoral immunity and upregulate antibody production to fight extracellular organisms. Overactivation of either pattern can cause disease, and either pathway can downregulate the other [105]. Th1 cells secrete large amounts of interferon- (IFN-) *γ*, IL-2, IL-3, granulocyte macrophage colony-stimulating factor, and a small amount of TNF. Th2 cells produce large amounts of IL-3, IL-4, IL-5, IL-6, and IL-10 and a small amount of TNF. Brandenberger et al. demonstrated that silica nanoparticles promote an adjuvant Th2/Th17 response in murine allergic airway disease [106]. Recently, Tomić et al. demonstrated that smaller gold nanoparticles (10 nm) have stronger inhibitory effects on maturation and antitumor functions of DCs, which were induced either by LPS or heat-killed tumor necrotic cells, compared to larger gold nanoparticles (50 nm). Gold nanoparticles (10 nm) can inhibit LPS-induced production of IL-12p70 by dendritic cells and potentiated Th2 polarization, while 50 nm gold nanoparticles promoted Th17 polarization [107]. The authors supposed that the size-dependent immunomodulatory effects of gold nanoparticles could be attributed to different mechanisms of their internalization, levels of accumulation, and intracellular distribution within DCs, leading to different modulation of maturational signaling. Furthermore, these results point to potential adverse effects of smaller gold nanoparticles if used in photo-thermal therapy and cancer diagnostics. The Th1 or Th2 responses elicited by APCs may be influenced by many factors, such as the maturation states of the APCs and routes of antigen uptake. Nanoparticle size plays a decisive role in determining whether antigens conjugated nanoparticles induce Th1 or Th2 immune responses [108]. Therefore, nanoparticle size may be a critical and fundamental parameter for induction of specific immunity in vaccine development. The precise selection of nanoparticle size for vaccination can influence the type 1/type 2 cytokine balance after one immunization, and this will be useful in the development of effective vaccines against common human pathogens. However, it is still unclear whether other different physical and chemical properties of nanoparticles, such as charge or chemical stability, can drive the T cell polarization.

### 2.7. Effects of Nanoparticles on B Cells

B cells are another type of lymphocytes in the adaptive immune system. B cells present unique surface receptor (B cell receptor) to bind with specific antigen. When B cell receptor binds with its specific antigen, antigen is delivered, degraded, and returned to surface bound with MHC class II. This antigen, MHC II complex, can be recognized by antigen-specific T helper cell. B cells receive an additional signal from a T helper cell, further differentiating into antibody-secreting B cells. It is reported that nanostructure of antigens is used to improve B cell antibody response [109]. Different kinds of synthetic nanoparticles are designed to carry antigens as effective vaccination system [101]. Temchura et al. recently reported that calcium phosphate (CaP) nanoparticles coated with protein antigens are promising vaccine candidates for induction humoral immunity [110]. In general, it is believed that nanoparticles did not result in the activation of B-cells, unless they were coated with the antigen. In contrast, it was also reported that iron oxide nanoparticles can compromise subsequent antigen-specific immune reaction, including antibody productions and T cell responses [111]. The effects of various metal-based nanoparticles on B cell functions are worthy to further and more comprehensive investigations and further to develop their potential applications.

### 2.8. Therapeutic Approach of Nanoparticles on Lymphoma

Lymphoma is a type of immune cell cancer occurring in B or T lymphocytes which divide faster than normal cells or live longer than they are supposed to. It was reported that the engineering nanoparticles have the potential to develop a nontoxic new treatment for lymphoma and other cancers which does not involve chemotherapy [112]. Yang et al. used gold nanoparticle combined with synthetic HDL (high-density lipoprotein) to trick B cell lymphoma, which prefers to eat HDL cholesterol. Once the B cell lymphoma cells start eating the gold nanoparticles (or artificial HDL particles), they get plugged up and can no longer feed on any more cholesterol. Deprived of B cell lymphoma's favorite food, the lymphoma cells essentially starve to death. The common treatments of lymphoma are chemotherapy, radiotherapy, or bone marrow transplantation. However, the chemotherapy has strong side effects, even leading to possible long term consequences such as infertility, second cancer risks, and lung damages. Promising and effective nanoparticles drugs may prevent occurrences of these side effects. While designing novel nanodrugs for cancer therapy, we should consider their molecular mechanisms; for example, Ag nanoparticles have been reported to have antiangiogenic ability [113]. Therefore, Ag nanoparticles are one of attractive and potential approaches to develop antitumor effect. Sriram et al. also demonstrated the antitumor activity of silver nanoparticles in Dalton's lymphoma ascites tumor model both* in vitro* and* in vivo* by activation of caspase-3 enzyme [114]. Moreover, nanodrugs are mainly developed according to their ability to distinguish between malignant and nonmalignant cells, making them a promising alternative to existing drugs. The targeting efficiency of nanoparticles can be accomplished by combining with RGD peptide [115] or antibody against specific tumor markers [116]. In a nutshell, nanoparticles may provide a new way to kill lymphoma without chemotherapy.

## 3. Conclusion and Future Perspectives

Nanoparticles can be used as vaccine carriers, adjuvants, and drug delivery vehicles to target specific inflammation-associated diseases or cancer. Nanoparticles, particularly noble metal nanoparticles, have considerable potential for biomedical applications, such as diagnostic assays, thermal ablation, and radiotherapy enhancement as well as drug and gene delivery. Currently, we are still challenged by limited knowledge of nanoparticle pharmacokinetics, biodistribution, and immunotoxicity.

The interactions of nanomaterials with the immune system have attracted increasing attention. The physicochemical properties of nanoparticles influence the immunological effects of nanoparticles. Comprehensive studies to explore the effects of physicochemical properties (such as size, shape, and charge) on the immunotoxicity of metal-based nanoparticles are still needed. Assessment of potential adverse effects on the immune system is also a critical component of the overall evaluation of nanodrug toxicity. Further mechanistic studies investigating nanoparticle immunomodulatory effects or inflammatory reactions are required to improve knowledge of the physicochemical properties of nanoparticles, which influences the immune system. A cooperation between materials science and immunology, immunobioengineering, is an emerging field which has great potential to develop prophylactic and therapeutic vaccine applicants.

## Figures and Tables

**Table 1:** Selected comparative *in vitro* and *in vivo* toxicity studies.

Nanoparticle rank for toxicity	Cell line(s)	Dose and time	Comments	References
Cu > Zn > Co > Sb > Ag > Ni > Fe > Zr > Al_2_O_3_ > TiO_2_ > CeO, low toxicity for W	Two human pulmonary cell lines (A549 and THP-1)	0.1–3300 *μ*g/mL, 3 and 24 h	MTT assay on THP-1 cell line exposed to NP for 24 h most sensitive experimental design	Lanone et al. [64]
ZnO > CeO_2_/TiO_2_	BEAS-2B	6.125–50 *μ*g/mL, 1–6 h	ZnO comparatively more toxic than TiO_2_ or CeO_2_ due to particle dissolution to Zn^2+^	George et al. [65]
ZnO > CeO_2_/TiO_2_	BEAS-2B and RAW264.7 macrophages	10–50 *μ*g/mL, 1–24 h	ZnO dissolution in endosomes CeO_2_ suppressed ROS production and TiO_2_ did not elicit protective or adverse effects	Xia et al. [66]
ZnO > Fe_2_O_3_ > TiO_2_/CeO_2_	Human mesothelioma and rodent fibroblast cell line	30 *μ*g/mL, 3–6 days	Human MSTO cells highly sensitive to Fe_2_O_3_	Brunner et al. [67]
ZnO > Fe > SiO_2_	L2 rat epithelial cells and rat primary alveolar macrophages and cocultures	0.0052–520 mg/cm^2^, 1–48 h	*In vivo* and *in vitro* measurements demonstrated little correlation	Sayes et al. [68]
ZnO > TiO_2_, Fe_3_O4, Al_2_O_3_,and CrO_3_	Neuro-2A cell line	10–200 *μ*g/mL, 2–72 h	ZnO was more toxic compared to other NPs	Jeng and Swanson [69]
CdCl_2_ > CdSO_4_ > ZnSO_4_ > ZnO > CuSO_4_ > ZnCl_2_ > V_2_O_5_ > CuCl_2_ > NiSO_4_ > NiCl_2_ > Fe_2_(SO_4_)_3_ > CrCl_2_ > VCl_2_ > CrK(SO_4_)_2_ > FeCl_2_	A549	0.005–5 mM, 2–48 h	RLE-6TN rat epithelia cells more sensitive than A549 cells	Riley et al. [70]
Ag > Fe_2_O_3_ > Al_2_O_3_ > ZrO_2_ > Si_3_N_4_ > TiO_2_ in RAW264.7 and ZrO_2_ >Al_2_O_3_/Fe_2_O_3_/Si_3_N_4_/Ag > TiO_2_ in THB-1 and A549	Murine alveolar macrophage (RAW264.7), human macrophage (THB-1), and human epithelial A549	5 *μ*g/mL, 48 h	THB-1 and A549 cells more sensitive than RAW264.7 and no correlation between specific surface area or NP morphology and toxicity	Soto et al. [71, 72]
Ag > MoO_3_ > Al/Fe_3_O_4_/TiO_2_	Rat cell line (BRL 3A)	5–25 *μ*g/mL, 24 h	Ag produces toxicity through oxidative stress	Hussain et al. [73]
Ag > Mn	PC-12 cells	1–100 *μ*g/mL, 24 h	Ag produced cell shrinkage and irregular membrane borders and Mn dose-dependently depleted dopamine	Hussain et al. [74]
Ag > NiO > TiO_2_	Murine macrophage cell line	5 *μ*g/mL, 48 h	Nanoparticles characterized as aggregates, caution on Ag	Soto et al. [75]
Ag > MoO_3_ > Al	Mouse spermatogonial stem cells	5–100 *μ*g/mL, 48 h	Concentration-dependent toxicity for all NPs tested	Braydich-Stolle et al. [76]
Cu and Mn > Al	PC-12 cells	10 *μ*g/mL, 24 h	Txnrd1, Gpx1, Th, Maoa, Park2, and Snca genes expression altered	Wang et al. [77]
VOSO_4_ > TiO_2_, SiO_2_, NiO, Fe_2_ O_3_, CeO_2_, and Al_2_O_3_	BEAS-2B	1–100 *μ*g/mL, 24 h	Manufactured pure oxides less toxic than natural particulate matter derived from soil dust and IL-6 secretion did not correlate with the generation of ROS in cell-free media	Veranth et al. [78]
Mn_3_O_4_ > Co_3_O_4_ > Fe_2_O_3_ > TiO_2_	Lung epithelial cells A549	30 *μ*g/mL, 4 h	Acellular ROS assay demonstrates catalytic conditions of NPs based on elemental composition	Limbach et al. [79]
Al > Al_2_O_3_	Rat alveolar macrophages	25–250 *μ*g/mL, 24 h	Phagocytosis hindered after exposure to Al NPs	Wagner et al. [80]

Nanoparticle(s)	Animal	Dose/route	Result	References

Ag	Rat	30–1000 mg/kg (subacute oral for 28 days)	Dose-dependent effect on alkaline phosphatase and cholesterol. Twofold more accumulation of NP in kidneys of female than male	Kim et al. [81]
Ag	Rat	1.73 × 10^4^/cm^3^ to 1.32 × 10^6^/cm^3^ (subacute inhalation, 6 h/day, 5 days/week for 4 weeks)	Liver histopathological effect but no effect in hematology and biochemical parameters	Ji et al. [82]
Ag	Zebrafish	5–100 *μ*g/mL (exposure, 72 h)	Dose-dependent toxicity in embryos Ag NP distributed in brain, heart, yolk, and blood of embryos	Asharani et al. [83]
Ag	Rat	NP was implanted intramuscularly for 7, 14, 30, 90, and 180 days	Inflammation	Chen et al. [84]
Ag	Mice	100–1000 mg/kg (acute oral)	Oxidative stress gene expression alterations	Rahman et al. [85]
Ag, Cu, and Al	Mice and rat	30–50 mg/kg (intravenous/intraperitoneal)	BBB penetration	Sharma [86]
Au	Mice	2 × 10^5^ PPB (oral for 7 days)	NP uptake occurred in the small intestine by persorption through single, degrading enterocytes extruded from a villus Smaller particles cross the GI tract more readily	Hillyer and Albrecht [87]
Cu	Zebrafish	0.25–1.5 mg/L (exposure, 48 h)	Biochemical, histopathological changes and alterations in gene expression	Griffitt et al. [88]
Cu	Mice	108–1080 mg/kg (acute oral)	NP-induced gravely toxicological effects and heavy injuries on kidney, liver, and spleen of treated mice	Chen et al. [89]
Fe_2_O_3_	Rat	0.8–20 mg/kg (inhalation)	Oxidative stress, inflammation, and pathology	Zhu et al. [90]
TiO_2_	Mice	5 g/kg (acute oral)	Biochemical and histopathological effects	Wang et al. [91]
SiO_2_ magnetic-NPs	Mice	25–100 mg/kg (intraperitoneal for 4 weeks)	NPs were detected in brain indicating BBB penetration	Kim et al. [92]

This table was reproduced from Schrand et al. [10].
